# Optic Nerve Neuroprotection in Glaucoma: A Narrative Review

**DOI:** 10.3390/jcm13082214

**Published:** 2024-04-11

**Authors:** Angela D’Angelo, Livio Vitiello, Filippo Lixi, Giulia Abbinante, Alessia Coppola, Vincenzo Gagliardi, Alfonso Pellegrino, Giuseppe Giannaccare

**Affiliations:** 1 Department of Medicine and Surgery, University of Naples “Federico II”, 80138 Naples, NA, Italy; dangeloangela99@gmail.com; 2 Eye Unit, “Luigi Curto” Hospital, Azienda Sanitaria Locale Salerno, 84035 Polla, SA, Italy; giulia.abbinante@gmail.com (G.A.); alessiacoppola330@gmail.com (A.C.); v.gagliardi@aslsalerno.it (V.G.); al.pellegrino@aslsalerno.it (A.P.); 3 Eye Clinic, Department of Surgical Sciences, University of Cagliari, 09124 Cagliari, CA, Italy; f.lixi0106@gmail.com (F.L.); giuseppe.giannaccare@gmail.com (G.G.)

**Keywords:** glaucoma, neurodegenerative diseases, neuroprotection, optic nerve

## Abstract

In recent years, researchers have been interested in neuroprotective therapies as a cutting-edge therapeutic strategy to treat neurodegenerative disorders by shielding the brain system from harmful events. Millions of individuals worldwide suffer from glaucoma, an ocular neurodegenerative disease characterized by gradual excavation of the optic nerve head, retinal axonal damage, and consequent visual loss. The pathology’s molecular cause is still mostly unknown, and the current treatments are not able to alter the disease’s natural progression. Thus, the modern approach to treating glaucoma consists of prescribing medications with neuroprotective properties, in line with the treatment strategy suggested for other neurodegenerative diseases. For this reason, several naturally derived compounds, including nicotinamide and citicoline, have been studied throughout time to try to improve glaucoma management by exploiting their neuroprotective properties. The purpose of this review is to examine the naturally derived compounds that are currently utilized in clinical practice for neuroprotection in glaucomatous patients based on scientific data, emphasizing these compounds’ pivotal mechanism of action as well as their proven therapeutic and neuroprotective benefits.

## 1. Introduction

Neurodegeneration is characterized by a gradual loss of neurons and associated processes (axons, dendrites, and synapses), together with a concurrent impairment of neuronal function [1]. Nowadays, glaucoma is recognized as a chronic neurodegenerative disease, affecting the entire visual pathway from the eye to the visual cortex. It is one of the leading causes of low vision in the elderly globally, and its effects, as a social health issue, are only going to get worse with time. Indeed, by 2040, it is predicted to have an impact on over 112 million individuals all over the world [2]. Considering its neurodegenerative nature, pharmacological approaches used in different degenerative brain disorders could also be beneficial in treating glaucoma and other optic neuropathies [3]. In particular, the death of retinal ganglion cells (RGCs) is one of the pivotal pathophysiological events in glaucoma [4]. For this reason, in recent years, neuroprotection has drawn increasing attention as a novel approach to stop or slow the development of morphological and functional glaucomatous impairments, particularly focusing on delaying the RGC degeneration. In fact, neuroprotection aims to preserve the structure and function of the neurons, trying to lower the rate of neuronal loss over the time [5].

For this reason, several molecules, including citicoline, have been tested over the years to try to improve the management of glaucoma by exploiting their neuroprotective properties [3]. However, the intricacy of the visual system’s anatomy and tissues, the timing of patient enrollment in clinical trials, the lack of valid endpoints, and the incomplete understanding of the main molecular basis of neurodegenerative diseases, including glaucoma, are some of the major factors that make it difficult to design effective neuroprotective strategies [6].

The aim of this review is to analyze the main naturally derived molecules currently used in clinical practice according to scientific evidence for neuroprotection in glaucomatous patients, focusing on their main mechanisms of action and their demonstrated therapeutic and neuroprotective effects, to provide some useful information to clinicians involved in the clinical management of this ocular neurodegenerative disease.

## 2. Main Naturally Derived Compounds with Neuroprotective Properties

### 2.1. Citicoline

Citicoline, also known as cytidine-5′-diphosphocholine or CDP-choline, is a mononucleotide composed of ribose, pyrophosphate, and cytosine [3]. It is a naturally occurring endogenous substance that acts as an intermediary in the synthesis of phosphatidylcholine, a crucial phospholipid in neuronal membranes. This occurs through the activation of biosynthesis pathways leading to the formation of structural phospholipids in neuronal membranes [4]. Citicoline increases the metabolism of cerebral structures, preventing phospholipid degradation and promoting an elevation in the levels of different neurotransmitters and neuromodulators in the central nervous system [7,8]. Citicoline has great neuroprotective properties mediated through various mechanisms. These include the maintenance of sphingomyelin and cardiolipin levels (essential for mitochondrial electron transport in the inner mitochondrial membrane), restoration of phosphatidylcholine levels, increased activity of glutathione reductase and glutathione synthesis, reduction of lipid peroxidation, and restoration of Na+/K+ ATPase activity [9]. Moreover, citicoline enhances the levels of acetylcholine, dopamine, noradrenaline, and serotonin in various brain regions [9], and it stimulates dopamine release in the retina [10].

Considering these properties, citicoline has been researched as a possible promising treatment for several neurological conditions, including glaucoma, brain ischemia, and Parkinson’s and Alzheimer’s diseases. [3,11,12,13].

Nowadays, citicoline is globally available as a dietary supplement and is used as a drug in many countries. It can be given through intravenous or oral administration, with very good bioavailability. Once absorbed, is quickly metabolized to cytidine and choline and distributed throughout the body; it crosses the blood–brain barrier and reaches the central nervous system, where it is incorporated into the membrane phospholipid fraction [14]. Citicoline demonstrates minimal toxicity, making it a safe molecule with few adverse effects. The only reported side effect is digestive intolerance following oral administration [15].

The proven daily therapeutic dosage of citicoline in humans is 500–2000 mg (7–28 mg/kg) [15], and it is also currently available for topical treatment and can be administered as eye drops to enhance patient compliance and adherence [3]. Citicoline appears to be involved in mitigating RGC dysfunction and preventing their apoptosis. Citicoline showed antiapoptotic effects on damaged RGCs and enhanced axon regeneration in a mouse retinal explant [16]. Moreover, several trials have also demonstrated the therapeutic effectiveness of citicoline therapy in glaucoma patients. The citicoline treatment for 2–8 years in glaucoma patients with moderate visual defects improved retinal function and neural conduction. The continuation of the treatment significantly slowed, stabilized, or even improved glaucomatous visual dysfunction [17]. Similarly, a study by Ottobelli et al. confirmed the enduring benefit of oral citicoline supplementation, suggesting that this intervention could considerably attenuate the glaucoma progression [18]. The role of citicoline was also investigated in association to other neuroprotective products. Melecchi and colleagues reported that citicoline combined with niacin was effective in restoring RGC activity in an animal model of induced ocular hypertension. Moreover, the combination of these substances showed better efficacy over each single compound in reducing inflammatory and oxidative stress markers and preserving mitochondrial function [19]. Similarly, Mastropasqua et al. described a synergistic effect of citicoline, coenzyme Q10, and vitamin B3, which improved mitochondrial activity, reduced inflammation and oxidation, and exhibited neuroprotective properties in vitro [20].

Research on citicoline’s role in glaucoma is expanding and, overall, the results consistently underscore citicoline as a safe compound with beneficial effects on visual function. However, further studies are essential to clarify the dose–response relationship and support the demonstrated clinical benefits of this interesting neuroprotective molecule.

### 2.2. Homotaurine

Homotaurine (also known as tramiprosate, 3-amino-1-propanesulfonic acid, or 3-APS) is a naturally sourced amino sulfonate compound identified in marine red algae, originally extracted from this source [21].

This compound is recognized for its potential to enhance the transmission of nervous impulses in the central nervous system and its neuromodulatory effects [22]. Homotaurine has the ability to interfere with various biological pathways in both in vitro and in vivo experimental models. It has been discovered to possess cytoprotective, neuroprotective, and neurotropic activities through a range of different mechanisms. It is also an analogue of GABA (4-aminobutyrate, γ-aminobutyric acid), thus exerting potent agonistic activity on GABA receptors, with a preference for GABA type A receptors. This characteristic contributes to its anti-nociceptive and analgesic activities, likely mediated by opioid and cholinergic mechanisms [23]. As a sulfur-containing amino acid, homotaurine could have a protective effect against cellular damage, especially in defense against oxidative damage to DNA caused by free radicals [24]. Additionally, it possesses the ability to prevent the formation of β-amyloid plaques, which are implicated in the apoptosis of neuronal cells and various neurodegenerative processes in the central nervous system [22]. This effect extends not only to the central nervous system but also to the ophthalmic field. The existing literature has brought attention to the potential involvement of amyloid in the induction of RGC apoptosis in experimental glaucoma models [25], suggesting that homotaurine could serve as a potentially effective treatment for glaucoma. In a recent study, it was found that the combined formulation of citicoline and homotaurine exhibits potent synergistic neuroprotective effects in cultured retinal cells, reducing the proapoptotic consequences linked to exposure to both glutamate and elevated glucose levels [26]. In another study, a randomized controlled multicentric trial demonstrated that the daily oral intake of the fixed combination of citicoline and homotaurine for 4 months led to an improvement in the function of inner retinal cells. This improvement occurred independently of intraocular pressure (IOP) reduction and positively impacted both the visual field and the perception of quality of life [27].

### 2.3. Coenzyme Q10

Coenzyme Q10 (CoQ10), also known as ubiquinone or ubidecarenone, is a 1,4-benzoquinone and Q represents the quinone chemical group. It is a naturally occurring hydrophobic molecule synthesized endogenously in every cell of animals and plants. Intracellular synthesis is the main source, but a small portion is introduced through the diet. The richest nutritional sources of CoQ10 are liver and other animal organs, as well as meat, fish, nuts, and some oils, while much lower levels can be found in dairy products, vegetables, fruits, and cereals [28]. CoQ10 is a ubiquitous compound essential for various functions associated with energy metabolism. It is an important cofactor of the mitochondrial electron transport chain, acting as a mobile electron and proton transporter from complex I (NADH: ubiquinone reductase) and complex II (succinate: ubiquinone reductase) to complex III (ubiquinone cytochrome c oxidase) in the inner mitochondrial membrane [29]. In addition, when CoQ10 turns from its oxidized form, ubiquinone, to the fully reduced form, ubiquinol, it acts as a potent lipid-soluble antioxidant and scavenger of free radicals. It directly eliminates free radicals or recycles and regenerates other antioxidants such as ascorbic acid (vitamin C) and tocopherol (vitamin E) [30]. In this way, it protects membrane lipids, proteins, and mitochondrial DNA from oxidative damage. Considering that dysfunctional energy metabolism and oxidative stress could be the main causes of the pathogenesis of many pathological conditions, including neurodegenerative diseases, CoQ10 has been utilized in the treatment of cardiac, neurologic, oncologic, and immunologic disorders [31,32]. The levels of CoQ10 decrease with age in the brain and various tissues in animals and humans. As a result, CoQ10 also plays an effective therapeutic role in age-related neurodegenerative disorders, such as Alzheimer’s and Parkinson’s disease, multiple sclerosis, epilepsy, depression, stroke, and glaucoma [32,33]. Due to its significant molecular weight and hydrophobic properties, the topical application of CoQ10 has poor intraocular penetration and limited bioavailability. Additionally, CoQ10 is a substrate of P-glycoprotein, an efflux membrane transporter expressed on RGCs and corneal epithelial cells. To avoid this, CoQ10 is often combined with vitamin E, which improves its bioavailability by inhibiting P-glycoprotein [34,35].

Evidence of mitochondrial dysfunction in neurodegenerative diseases comes from animal models, studies on patients’ mitochondria, and assessments of oxidative stress markers. Therefore, the potential neuroprotective effect of CoQ10 has been demonstrated in several in vitro studies and animal models [36]. In glaucoma-related models, topical treatment with CoQ10 or its integration into the diet has demonstrated the ability to protect RGCs against oxidative stress and to promote RGC survival by inhibiting RGC apoptosis [37,38]. In a mechanical optic nerve injury rat model, topical treatment with a combination of CoQ10 and vitamin E increased the number of RGCs, inhibiting apoptosis and astrocyte and microglial cell activation [39]. Furthermore, several studies highlighted the neuroprotective effect of CoQ10 on the retina. In fact, topical treatment with CoQ10 in a rat model of transient IOP elevation reduced extracellular glutamate levels, decreased retinal damage, and avoided apoptotic cell death [40]. In a mouse model of retinal ischemia/reperfusion injury, dietary supplementation with ubiquinol (the reduced form of CoQ10) enhanced the survival of RGCs by preventing cell death mediated by apoptosis. Additionally, it inhibited microglial cell and astrocyte activation in the retina [41]. In addition, in a 12-month clinical trial, vitamin E and CoQ10 eye drops improved inner retinal function and enhanced visual cortical responses in primary open-angle glaucoma patients [42].

### 2.4. Epigallocatechin-3-Gallate

Epigallocatechin-3-gallate (EGCG) is a type of catechin, a polyphenolic compound highly abundant in green tea, comprising 50–70% of its catechins. Due to its prominence in tea, much of the research on green tea centers around EGCG. However, clinical use of EGCG still faces several challenges. When taken orally, it has low bioavailability, estimated to be between 0.1% and 0.3% in both rats and humans. It enters the bloodstream at a very low micromolar concentration and disappears from the plasma within several hours (<8 h) due to fast metabolism (glucuronidation, methylation, and sulfation) and microbial metabolism. For this reason, finding effective ways to deliver EGCG to specific target sites remains a complex issue [43,44]. Several studies have demonstrated that EGCG is a potent antioxidant with multifunctional properties, including anti-inflammatory [45] and vasodilator effects, which all contribute to its neuroprotective action. Animal studies have demonstrated that EGCG also possesses anti-aging properties by functioning as a scavenger for free radicals [46]. Moreover, cancer chemopreventive/chemotherapeutic effects have also been demonstrated [47].

In an interventional study involving a mouse model with optic nerve crush, systemic treatment with EGCG demonstrated protective effects on RGCs after this event, suggesting that EGCG could be a promising therapeutic agent for optic nerve diseases, including glaucoma [48].

### 2.5. Vitamins

Vitamins constitute a heterogeneous group of organic compounds classified as micronutrients, as they are required by the body in limited quantities, typically in the range of milligrams or micrograms. Vitamins are generally classified based on their chemical solubility properties: fat-soluble vitamins are A, D, E, and K and water-soluble vitamins include B complex vitamins and vitamin C. Vitamins play a crucial role in regulating numerous chemical reactions within our body and can act as coenzymes, biological antioxidants, cofactors in redox reactions, or hormones. The primary mechanism by which vitamins may have neuroprotective benefits is probably due to their antioxidant activity [49,50,51,52].

Several studies have explored the relationship between dietary or supplement intake of antioxidants and glaucoma risk, with controversial results.

In a cross-sectional study conducted as part of the National Health and Nutrition Examination Survey in the United States during 2005–2006, involving 2912 participants, Wang and colleagues focused on the association between glaucoma prevalence and supplement intake, as well as serum levels of vitamins A, C, and E. The study found no association between either supplementary consumption or serum levels of vitamins A and E with glaucoma prevalence. Interestingly, both low- and high-dose supplementary consumption of vitamin C were associated with decreased odds of glaucoma, while serum levels of vitamin C did not correlate with glaucoma prevalence [53]. A systematic review found that blood levels of vitamins (A, B complex, C, D, and E) did not show an association with open-angle glaucoma, but this study highlighted a positive association between the dietary intake of vitamins A and C and open-angle glaucoma [54]. Similarly, a meta-analysis did not find a significative association between serum vitamin B6, vitamin B12, and vitamin D levels and different types of glaucoma [55].

In a prospective study, which included 116,484 participants aged ≥ 40 years, no significant association was found between dietary intake of antioxidant vitamins (A, C, and E) and carotenoids and the risk of primary open-angle glaucoma over an average follow-up period of 9.1 years [56]. Conversely, in a cross-sectional study involving 1155 participants, increased consumption of specific fruits and vegetables containing vitamins A, B2, C, and carotenoids was linked to a reduced glaucoma risk in old women [57]. In The Rotterdam Study, a prospective study involving a cohort of 3502 participants aged 55 years and older, no significant association between the intake of most evaluated antioxidant carotenoids and vitamins (C and E) and open-angle glaucoma was found. However, compared to the group with a low consumption, those with a high intake of vitamin B1 and retinol equivalents showed approximately a twofold decreased incidence of open-angle glaucoma [58]. In a randomized controlled trial carried out on older adults, the use of oral antioxidant supplementation, which included B-group vitamins;, vitamins A, C, and E; carotenoids; and antioxidant minerals, with or without omega-3 fatty acids, over a 2-year follow-up did not prevent visual field loss or thinning of the retinal nerve fiber layer or ganglion cell complex. This suggests that such supplementation does not appear to be beneficial as an adjuvant treatment for mild to moderate primary open-angle glaucoma in the short term [59].

Considering all these findings, the role of vitamins as neuroprotective agents in glaucoma management is still controversial.

#### 2.5.1. Vitamin B1

Vitamin B1, also known as thiamin, is an essential cofactor, indispensable for the normal growth and development of the body, playing a crucial role in various biochemical and physiological processes. Vitamin B1 is abundant in foods such as meat, whole grains, eggs, fish, legumes, and nuts [60]. Vitamin B1 deficiency rarely manifests as optic neuropathy, typically presenting as bilateral, severe involvement with associated optic disc swelling [61,62]. The Rotterdam Study suggested a protective effect of thiamine against open-angle glaucoma [58], whereas the study by Giaconi et al. did not report any association between vitamin B1 and open-angle glaucoma [63].

#### 2.5.2. Vitamin B2

Riboflavin, also known as vitamin B2, plays a vital role in cellular processes, serving as a component of coenzymes like flavin mononucleotide and flavin adenine dinucleotide involved in electron transport. Rich sources of riboflavin include poultry, fish, eggs, dairy products, and various plant foods [64]. A study by Coleman et al. observed a decreased risk of glaucoma diagnosis in women consuming at least 2 mg/day of vitamin B2 from natural food sources [57].

#### 2.5.3. Vitamin B3

Nicotinamide is a water-soluble amide that, together with nicotinic acid, forms the niacin and/or vitamin B3 complex. It is a precursor to nicotinamide adenine dinucleotide (NAD+), playing a crucial role in essential cellular functions. As a key component of the glycolysis pathway, nicotinamide contributes to NAD+ production for ATP generation, influencing cellular energetics and various metabolic processes [65]. NAD+ is a crucial coenzyme in hydride-transfer enzymes, essential for energy production and the synthesis of fatty acids, cholesterol, and steroids. It participates in oxidation–reduction reactions as a hydride donor (NADH and NADPH) and acceptor (NAD+ and NADP). NAD+ primarily acts in catabolic reactions, breaking down carbohydrates, fats, proteins, and alcohol for energy, while NADP is involved in anabolic reactions, contributing to the synthesis of cellular macromolecules [66].

Meat, eggs, fish, dairy, coffee, tea, and niacin-fortified cereals are particularly rich sources of nicotinamide, while it is found in lesser amounts in vegetables. Nicotinamide can also be produced from dietary tryptophan, which is an essential amino acid [67]. Nicotinamide, classified as a food additive product rather than a pharmaceutical product, exhibits good bioavailability when administered orally. It distributes effectively across all body tissues, undergoes metabolism in the liver, and is excreted through the kidneys. The recommended dietary intake of nicotinamide is approximately 15 mg/day. Adverse effects from excessive intake, even at pharmacologically high doses, are uncommon. [68]. Nicotinamide also has anti-inflammatory effects; photoprotective effects on the skin; and reduces pigmentation, wrinkles, ultra-violet induced immunosuppression, and sebum production [69]. Moreover, nicotinamide has long been linked to neuronal development, survival, and function in the central nervous system. It contributes to neuronal death as well as neuroprotection, and a number of studies point to its significance in neurological disorders and neurodegenerative illnesses [70].

The enzyme nicotinamide phosphoribosyl transferase is responsible for synthesizing nicotinamide mononucleotide from nicotinamide. Its involvement in the metabolic pathway for NAD biosynthesis suggests its importance in cells sensitive to declining NAD levels, particularly neurons [71]. Changes in NAD homeostasis have been observed with aging [72], suggesting that nicotinamide may play a crucial role in neuronal maturation and neuroprotection by influencing NAD+ levels within neurons. Furthermore, nicotinamide contributes to DNA stability and preserves membrane integrity, preventing cellular injury, phagocytosis, apoptosis, and the formation of vascular clots [73]. The multitude of intracellular systems affected by nicotinamide levels complicates the identification of precise mechanisms of action for this dietary metabolite. However, it seems that the pivotal way in which nicotinamide might act is by restoring ATP levels in neurons. Furthermore, it seems to be implicated in three key neurodegenerative conditions: Alzheimer’s, Parkinson’s, and Huntington’s disease [74,75,76]. In the ophthalmic field, a nicotinamide-supplemented diet decreased mitochondrial susceptibility and greatly prevented RGCs from degenerating, according to research on mice [77,78].

In a recent study, a cohort of individuals with primary open-angle glaucoma showed a notably lower concentration of nicotinamide compared to the control group [79]. In addition, in a crossover, double-masked, randomized clinical trial involving 57 participants with glaucoma under IOP-lowering medications, oral nicotinamide supplementation resulted in an early enhancement of inner retinal function [80]. All these findings seem to suggest that nicotinamide could represent a promising molecule for neuroprotection in glaucoma.

#### 2.5.4. Vitamin B6, Vitamin B9, and Vitamin B12

Vitamin B6 comprises a group of six water-soluble chemical compounds, and pyridoxal phosphate is the active form. B6 is a vitamin found in poultry, fish, and plant-based foods. Pyridoxal phosphate acts as a cofactor for approximately 160 reactions in the body, including gluconeogenesis and glycogenolysis to amino acid and lipids biosynthesis and metabolism [81]. Furthermore, vitamin B6 is involved in regulating homocysteine levels, an amino acid also associated with oxidative stress and apoptosis in RGCs [82].

Vitamin B9, also known as folic acid or folate, is essential for the synthesis of DNA and RNA, and plays a key role in the breakdown of homocysteine. Good sources of vitamin B9 include plants foods, such as leafy greens and legumes. Insufficient folate in adults is linked to cognitive decline and visual system complications, including nutritional amblyopia and optic disc disorders. In fact, low serum folate correlates with nutritional optic neuropathy, leading to gradual visual loss and symptoms like central scotoma and altered color perception [62,83].

Vitamin B12, or cobalamin, is obtained only through the intake of animal-sourced foods, such as eggs, fish, and meat. Vitamin B12 is essential in the metabolism of carbohydrates, lipids, and proteins, DNA synthesis, hematopoiesis, and the maintenance of the integrity of the peripheral and central nervous systems. The primary cause of vitamin B12 deficiency is prolonged dietary deprivation without adequate supplementation [84]. Vitamin B12 deficiency can lead to pernicious anemia, high homocysteine levels, and damage to the nervous system. Optic neuropathy may occur before hematologic abnormalities and be the first sign of a cobalamin deficit. [62]. A study involving patients with open-angle glaucoma exhibited significantly lower serum levels of vitamin B12 compared to controls [85]. On the other hand, other studies did not report significant differences in plasma levels of vitamin B6 or serum levels of vitamins B9 and B12 between open-angle glaucoma patients and controls [86,87]. However, lower levels of vitamin B9 and B12 are significantly linked to higher homocysteine levels, which has been associated with oxidative stress and apoptosis in RGCs. In fact, increased levels of homocysteine have been reported in the aqueous humor and plasma of patients with primary open-angle glaucoma. In addition, reduced levels of vitamin B6, B9, and B12 were also linked to increased homocysteine levels in individuals with pseudoexfoliation glaucoma [88].

#### 2.5.5. Vitamin C

Vitamin C, or ascorbic acid, is a potent antioxidant exerting beneficial effects on redox oxidative pathways, inflammaging, endothelial integrity, and lipoprotein metabolism. Vitamin C is synthesized from glucose in the liver of many mammalian species; however, humans have evolutionarily lost this synthetic ability, making it necessary to obtain vitamin C through the diet [89], and fruits and vegetables are the main sources of vitamin C. Concerning its neuroprotective properties, the studies investigating the association between blood levels of vitamin C and glaucoma showed conflicting results. In patients with normal tension glaucoma, lower serum levels of vitamin C were reported compared to controls [90], but in patients with open-angle glaucoma no significant differences were observed [91]. Conversely, a cross-sectional study involving 2912 participants aged > 40 highlighted weak evidence that supplemental vitamin C intake might possibly be associated with a decreased risk of glaucoma [53].

#### 2.5.6. Vitamin A

Vitamin A (retinol) acts as a significant dietary antioxidant and is abundant in various sources, including dairy products, fish, meat, and plants. The biological activity of vitamin A is also exhibited by carotenoids, which act as provitamin A. Among these, β-carotene is the most active. β-carotene is a potent antioxidant, reducing the accumulation of reactive oxygen species, including hydrogen peroxide and lipid peroxide radicals [92]. Because β-carotene may reduce oxidative stress and counteract lipid peroxidation, it plays a major role in brain-related disorders [93]. Particularly, in brain diseases linked to reactive oxygen species, therapy with β-carotene may decrease neuronal loss since oxidative stress strongly contributes to neuronal death during neurotoxicity [94].

Five studies have examined the relationship between blood vitamin A levels and glaucoma, all showing conflicting results. In fact, one study reported higher vitamin A levels in patients with primary open-angle glaucoma compared to those with normal tension glaucoma [86], while another study similarly found higher levels compared to controls [95]. Conversely, three other studies did not identify significant differences in vitamin A concentration between individuals with glaucoma and healthy subjects [53,90,96].

Despite these conflicting results, The Rotterdam Study meta-analysis has found that individuals with a high intake of retinol equivalents have approximately half the risk of developing open-angle glaucoma compared to those with a low intake of these nutrients [58], suggesting a potential favorable association of dietary retinol intake in patients with open-angle glaucoma.

#### 2.5.7. Vitamin E

Vitamin E is a fat-soluble vitamin with eight identified isoforms that include four tocopherols and four tocotrienols designated as α-, β-, γ-, and δ-. The most studied isoform is α-tocopherol (αT). It is a potent antioxidant agent against oxidative stress damage caused by free radicals, preventing the oxidation of polyunsaturated fatty acids in cell membranes by donating hydrogen from the phenolic group on the chromanol ring. All forms of vitamin E have robust antioxidant activities, thanks to their similar phenolic moieties [97]. Vitamin E in its natural state is produced by plants, with αT being primarily concentrated in certain seeds. Notably, αT is abundantly found in peanuts, almonds, and sunflower seeds. Consequently, this form of vitamin E is prevalent in various food oils such as corn, soybean, and peanut oil [98]. Vitamin E is an essential micronutrient and in the intestinal tract, dietary tocopherols and tocotrienols are absorbed with dietary fats. In the liver, the αT isoform has a greater affinity for the αT transfer protein, which facilitates the incorporation of αT into lipoproteins. This process promotes the transportation of vitamin E to various tissues through circulation [99]. The current recommended daily intake of vitamin E for adults is set at 15 mg, for both males and females. Vitamin E plays a crucial role as an antioxidant in various tissues, including the eye. In fact, in rat models with increased IOP, those with a vitamin-E-deficient diet had significantly higher RGC death due to increased lipid peroxidation compared to rats following a diet containing vitamin E [100]. In humans, the reduction of glaucoma progression was observed with daily vitamin E integration [101], in addition to a neuroprotective effect of αT oral supplement against glaucomatous damage [102].

### 2.6. Forskolin

Forskolin is a diterpene abundant in the leaves, roots, and tubers of Coleus forskohlii, a medicinal plant native to India and Southeast Asia. It is a natural active compound that acts as a second messenger, stimulating cyclic adenosine monophosphate (cAMP) through direct activation of adenylate cyclase. In cells treated with forskolin, the intracellular concentration of the second messenger cAMP increases rapidly [103]. Several in vitro and in vivo studies highlight forskolin as a neuroprotective agent, considering its efficacy in lowering IOP in animals and humans [104,105] as well as its protective effect on RGCs against insults associated with glaucoma [106]. Therefore, forskolin may exert beneficial indirect neuroprotective effects on RGCs by reducing the IOP. In a double-blind, randomized, controlled trial, patients with primary open-angle glaucoma who were treated with forskolin 1% eye drops (two drops three times a day) for 4 weeks exhibited a notable reduction in IOP [107]. This could be explained by the hypotensive effect of forskolin, which leads to decreased aqueous humor accumulation [52]. In another clinical study, a dietary supplement containing forskolin given to patients with primary open-angle glaucoma was able to reduce IOP and enhance their pattern electroretinogram amplitude. This could suggest a positive impact on the survival and/or function of RGCs [108].

It seems that some of neuroprotective effects of forskolin are mediated by the activation and enhancement of neurotrophins’ activity. Meyer-Franke et al. showed that forskolin added to a culture medium containing brain-derived neurotrophic factor (BDNF), ciliary-derived neurotrophic factor (CTNF), and insulin-like growth factor-1 (IGF-1) increased the RGC lifespan [106]. Similarly, when included in a combined treatment with BDNF and CTNF, forskolin significantly enhanced the survival of axotomized RGCs in the cat retina [109].

In animal studies, a dietary supplementation of forskolin, homotaurine, spearmint, and vitamins B showed protect effects against RGC loss in rodent models of optic nerve injury [110] and glaucoma [111]. The forskolin supplement mixture was able to reduce inflammatory cytokines, leading to decreased apoptotic markers, sparing RGCs and preserving visual function, without impacting IOP in glaucomatous rodents [111].

### 2.7. Ribes Nigrum

Ribes nigrum, commonly known as blackcurrant, is a plant belonging to the Grossulariaceae family that contains 160 species, native to Europe and Asian Russia. Some species of Ribes have been employed in traditional and local medicine for the treatment of glaucoma, cardiovascular disease, hepatitis, hyperlipidemia, hypertension, and other health issues [112]. Blackcurrant is a good source of polyphenols, containing four different types of anthocyanins, which are the subject of research in studies on glaucoma progression. In a randomized, placebo-controlled study, Ohguro and colleagues investigated the influence of the blackcurrant anthocyanins (BCACs) on the disease progression of open-angle glaucoma in 38 patients treated using antiglaucoma drops. For a period of 24 months, participants were administered BCACs (50 mg/day, n = 19) or placebos (n = 19) orally once a day. The trial results showed that the BCAC-treated group exhibited a significant improvement in ocular blood flow and in the visual field, whereas no significant changes were observed in systemic and ocular conditions, including IOP [113]. In another study by Ohguro et al., the effects of oral administration of BCACs on IOP were investigated in both healthy subjects and patients with glaucoma. A double-blind, placebo-controlled crossover study was conducted with 12 healthy subjects treated once daily with oral BCACs (50 mg) and 21 primary open-angle glaucoma patients (BCACs, n = 12; placebo, n = 9) treated with a single antiglaucomatous drug. They observed a significative decrease in mean IOP values (at 4 weeks, *p* = 0.039), not only in healthy participants but also in glaucoma patients using BCACs (*p* = 0.027) over 2 years [114]. The results of these studies seem to suggest that the administration of BCACs may be a safe and promising supplement for healthy subjects and glaucomatous patients already treated with antiglaucomatous drugs.

### 2.8. Berberine

Berberine is a quaternary ammonium salt classified within the benzylisoquinoline alkaloids. It occurs natural in some plants of the *Berberis* genus, typically in the roots, rhizomes, stems, and bark. Berberine is widely utilized in various traditional medical systems, including Ayurvedic, Chinese, and Iranian medicine [115]. Berberine, exhibiting a variety of pharmacological activities, shows anti-heart failure, antioxidant, antimicrobial, anti-inflammatory, hypocholesterolemic, antitumor, and immunomodulatory properties [116,117,118,119]. Although its clinical uses are limited due to its poor solubility and bioavailability (less than 1%) [120], ongoing preclinical studies are driving exploration towards new potential applications. In recent years, many studies have investigated the role of berberine in central nervous system diseases. These investigations have revealed that berberine can effectively cross the blood–brain barrier, exerting positive effects on brain functions [121,122,123]. Berberine has demonstrated significant neuroprotective properties in studies carried out in vitro and on animal models against drug- and toxin-induced neurotoxicity, ischemia-reperfusion damage, and chronic neurodegenerative conditions such as Alzheimer’s and Parkinson’s diseases, depression, schizophrenia, epilepsy, and anxiety [122,124,125,126]. The neuroprotective effects of berberine are mediated by intricate molecular mechanisms that involve several biological functions, including antioxidant, anti-inflammatory, and antiapoptotic actions [127]. Currently, human studies reporting the pharmacological effects of berberine on neurodegenerative diseases are limited. Moreover, most studies on the use of berberine in treating neurodegenerative diseases have focused on Alzheimer’s and Parkinson’s diseases [128], with only a few involving other conditions. Therefore, its mechanism of action has been explored in vitro and in animal models, but much remains to be clarified in humans. Berberine could be a valuable potential therapeutic target for various neurodegenerative diseases, including glaucoma, but further research is still needed to fully understand the bioavailability, efficacy, and dosage of berberine in clinical studies.

### 2.9. Ginkgo Biloba

Ginkgo Biloba is a tree native to East Asia and belongs to the *Gymnosperm* species. It is a very ancient tree, first appearing approximately 250 million years ago, considered a “living fossil”. These trees have been used in traditional Chinese and Japanese medicine for centuries, with ginkgo seeds recognized for their therapeutic properties, while the leaves were commonly used in teas for medicinal purposes [129].

The plant’s composition includes bioactive compounds like flavonoids (e.g., quercetin and kaempferol), bioflavonoids, organic acids (e.g., ginkgolic acid), and terpene lactones (e.g., ginkgolides A, B, and C), expanding its utility across several biological systems. Most randomized, controlled trials utilize the standardized extract of Ginkgo biloba (GBE) leaves, EGb761. This extract, containing flavonoids (24%), terpene lactones (6%), and a low concentration of ginkgolic acids (0.0005%), has been shown to support age-related conditions including neurodegenerative disorders, cognitive decline, and glaucoma [130]. Its potential use is justified by its demonstrated neuroprotective and antioxidant properties, along with its ability to increase blood flow through vasodilation and reduce blood viscosity [131]. For glaucoma management, the effects of GBE on RGCs have been predominantly studied in animal models. Two studies have demonstrated that administering GBE daily for 4 weeks after an optic nerve crush injury led to increased survival rates of RGCs [132,133]. In another study conducted in vivo, the impact of GBE on increased IOP and RGC density was also evaluated, showing that both pretreatment and early posttreatment with EGb761 effectively exhibited neuroprotective effects in a rat model of chronic glaucoma [134]. Clinical studies involving GBE focused on two distinct outcomes: enhancement of blood flow or improvement in the visual field. In studies focusing on blood flow improvements, Park et al. found increased peripapillary blood flow in normal tension glaucoma patients after 4 weeks of GBE (80 mg twice daily) oral administration [135]. Similarly, the effects of GBE on ocular blood flow were affirmed in open angle glaucoma patients treated for 4 weeks with an antioxidant dietary supplement containing 120 mg/day of GBE [136]. On the other hand, there are conflicting results in studies regarding the effect of GBE on visual field outcomes [130]. Quaranta et al. carried out a study in Italy involving 27 patients with normal tension glaucoma, assessing visual field outcomes following GBE supplementation. Significant improvements were observed in visual field indices with 40 mg GBE three times/daily for 4 weeks compared to a placebo, suggesting potential benefits on retinal sensitivity and cognitive function. However, these effects were only temporary and did not persist after the washout period [137]. Another study also investigated GBE effects on visual field and contrast sensitivity in 28 Chinese patients with normal tension glaucoma but found no significant differences compared to a placebo [138]. Given the inconsistent findings in the existing literature, further research is warranted to determine the efficacy of GBE in glaucoma treatment and management. Additionally, it is also important to note that none of the current studies on GBE address the treatment and management of primary open-angle glaucoma, which is the most common clinical form.

## 3. Discussion

This review evaluated the neuroprotective properties of several nutrients in contemporary ophthalmology and medicine, as summarized in Table 1 and Table 2. Most of the molecules analyzed in this review carry out their neuroprotective action through a predominantly antioxidant action, protecting RGCs from oxidative stress, thus reducing their apoptosis and improving their visual function. The increasing prevalence of these dietary supplements in the published scientific literature heralds a new era in the treatment of many medical conditions, such as glaucoma, neurodegenerative diseases, and other ocular disorders. Nutrient-rich substances are an appealing inclusive choice in medical therapy because of their low cost, simple absorption, and lack of major negative effects when taken as prescribed. These supplements undoubtedly cannot take the place of conventional medical and surgical care, but they can have a synergistic beneficial impact that increases the effectiveness of gold-standard therapies and helps stabilize the patient’s overall health.

Furthermore, it is interesting to note how some of the molecules discussed in this review, in addition to their properties already analyzed, could have a further neuroprotective effect by stimulating the metabolic pathway of vascular endothelial growth factor (VEGF). In fact, it has been demonstrated in vitro and in animal models that coenzyme Q10, forskolin, and berberine can promote angiogenesis in cerebral ischemic areas [139,140,141]. However, to date, no in vivo clinical studies have yet been carried out on human models and, in particular, for glaucoma.

Another interesting aspect to consider about these molecules, in addition to the aforesaid therapeutic and neuroprotective effects, is their safe profile, which could make them easily and further usable in clinical practice. In fact, to date, only citicoline, homotaurine, coenzyme Q10, and Gingko biloba have been shown to have minimal side effects, mainly linked to digestive intolerance and at high doses. Furthermore, some of the discussed compounds, such as coenzyme Q10, forskolin, and Gingko biloba, can interfere with the action of some drugs, such as antihypertensive, antiplatelet, and anticoagulant drugs [142,143].

In addition, given the demonstrated effect of forskolin and Ribes nigrum on IOP reduction, it could be challenging to carry out clinical studies to demonstrate the effect of these two molecules or other ones in managing the IOP increase following intravitreal injections [144,145,146], since, to date, there are no data published in the scientific literature.

In Table 3, we summarized other minor naturally-derived molecules with potential neuroprotective properties, but future studies are needed to better understand their potential role and use in clinical practice.

This narrative review has some limitations including its narrative and non-systematic nature and having used a single scientific database (PubMed) for its drafting. Furthermore, in this review, only some of the main naturally derived molecules currently utilized in clinical practice for neuroprotection in case of glaucoma have been covered.

## 4. Conclusions

In conclusion, the treatment of neurodegenerative diseases, including glaucoma, may benefit from the integrative use of antioxidants, vitamins, organic compounds, and micronutrients as IOP-independent techniques [156]. Nonetheless, to fully use the neuroprotective qualities of these supplements as adjuvant therapeutic alternatives in treatment regimens, further research and even large-scale multicenter clinical studies are consistently needed to confirm their efficacy. Lastly, it is critical to remember that therapeutic medications in the form of eye drops, such as prostaglandin analogs and beta blockers, remain the first-choice therapeutic for glaucoma [157].

## Figures and Tables

**Table 1:** Summary of the main effects of the discussed neuroprotective molecules.

Active Compounds	Daily Therapeutic Dosage	Prescription Duration	Neuroprotective Effects	Administration	Bioavailability	Adverse Effect and Contraindications
Citicoline	500–2000 mg	2 weeks–4 months	Possible mitigation of RGC dysfunction and prevention of their apoptosis Improved retinal function and neural conduction Improved glaucomatous visual dysfunction	Oral Eye drops	High	Minimal toxicity, predominantly digestive intolerance
Homotaurine	50–100 g	2 weeks–4 months	Possible mitigation of RGC dysfunction and prevention of their apoptosis Improved retinal function	Oral	Moderate	Minimal toxicity, predominantly digestive intolerance
Coenzyme Q10	90–200 mg	Weeks–months	Protect RGCs against oxidative stress Promote RGC survival by inhibiting RGC apoptosis Enhance visual cortical responses in OAG	Oral Eye drops	Moderate/high, depending on the formulation	Minimal toxicity, predominantly digestive intolerance May increase the metabolism of warfarin May cause an excessive decrease in blood pressure when taken together with antihypertensive drugs May reduce the effectiveness of some pro-oxidant chemotherapy treatments
Forskolin	20–50 mg	Weeks–months	Decrease IOP Protective effect on RGCs	Oral Eye drops	Not available	Limited data May interact with antihypertensive drugs
Epigallocatechin-3-gallate	300 mg	Weeks–months	Antioxidant activity Protective effects on RGCs after optic nerve crush	Oral	Very Low	Limited data
Ribes nigrum	50 mg	Weeks–months	Improvement in ocular blood flow and in the visual field Decrease in mean IOP values	Oral	Not available	Limited data
Berberine	500–1500 mg	Weeks–months	Not fully demonstrated	Oral	Very low	Limited data
Ginkgo Biloba	80–600 mg	Weeks–months	Antioxidant activity Anti-inflammatory effects Increase ocular blood flow	Oral	Moderate/High	Minimal toxicity, predominantly digestive intolerance May cause bleeding with concomitant use of antiplatelet or anticoagulant drugs

RGCs: retinal ganglion cells; IOP: intraocular pressure; OAG: open angle glaucoma.

**Table 2:** Summary of the main neuroprotective effects of vitamins.

Vitamin	Vitamer Chemical Name	Recommended Daily Intake for Adults *	Sources	Neuroprotective Effects
B1	Thiamin	1.2 mg for man 1.1 mg for women	Cereal grain, meat, egg, fish, legume, nut	Possible protective effect against open-angle glaucoma
B2	Riboflavin	1.3 mg for man 1.1 mg for women	Poultry, fish, egg, dairy products, plant foods	Associated with lower risk of glaucoma diagnosis
B3	Niacin	16 mg for man 14 mg for women	Meat, egg, fish, dairy, coffee, tea	Protect retinal ganglion cells from degeneration Enhance inner retinal function
B6 B9 B12	Pyridoxin Folate Cobalamine	1–1.7 mg 400 mcg 2.4 mcg	Poultry, fish, plant-based foods Dark green leafy vegetables, nut, avocado Meat, fish, eggs	Regulators of homocysteine levels
C	Ascorbic acid	90 mg for men 75 mg for women	Fruits and vegetables	Antioxidant activity associated with lower risk of glaucoma diagnosis
A	Retinol	900 mcg 700 mcg	Carrot, tomato, butter, cream cheese, egg, fish	Antioxidant activity Associated with lower risk of open-angle glaucoma diagnosis
E	Tocopherol	15 mg	Peanuts, almonds, sunflower seeds	Antioxidant activity Associated with lower progression of glaucoma

* Institute of Medicine (US) Standing Committee on the Scientific Evaluation of Dietary Reference Intakes and its Panel on Folate, Other B Vitamins, and Choline. *Dietary Reference Intakes for Thiamin, Riboflavin, Niacin, Vitamin B6, Folate, Vitamin B12, Pantothenic Acid, Biotin, and Choline*. Washington, DC: National Academies Press (US); 1998.

**Table 3:** Summary of minor plant-derived neuroprotective compounds.

Active Compounds	Daily Therapeutic Dosage	Neuroprotective Effects	Administration	Bioavailability	Adverse Effect and Contraindications
Crocetin [147]	In rats, 50–100 mg/kg	Antioxidant activity ROS scavenger	Oral	Low	No adverse effects demonstrated No adverse effects are available in long-term administration
Hesperidin [148,149]	50 mg	Antioxidant activity Possible protection of RGCs against oxidative stress Possible prevention of RGCs’ death	Oral Intravitreal	Low	Low toxicity at a wide range of doses
Lycium barbarum [150]	In rats, 1 mg/kg	In animal model Antioxidant activity Anti-inflammatory effects Possible prevention of RGCs’ loss Preservation of retinal structure and function	Oral	Not available	No adverse effects demonstrated Possible interaction with warfarin
Tamarindus Indica [151,152]	In rats, 100–5000 mg/kg	Antioxidant activity	Oral	Not available	No toxic reported Potentially increases the bioavailability of aspirin
Resveratrol [153,154]	In rats, 20–250 mg/kg	Potential prevention retinal damage and RGC apoptosis Antioxidant activity Anti-inflammatory effects	Oral Intragastric Intraperitoneal	Low	No adverse effects demonstrated Alteration or inhibition of CYP3A4 enzyme activity May interact with blood thinners like warfarin, increasing the risk of bleeding
Scutellaria baicalensis Georgi [155]		Only in animal models in vitro Blood pressure level reduction Antioxidative effects Anti-inflammatory effects	Intraperitoneal	Not available	Limited data
Vaccinium myrtillus [156]	In rats 100–500 mg/kg	Blood pressure level reduction Strong antioxidative effects Anti-inflammatory effects	Oral	Not available	Rare allergic reaction

## Data Availability

The data can be shared upon request.
